# Should Burden of Disease Estimates Include Cannabis Use as a Risk Factor for Psychosis?

**DOI:** 10.1371/journal.pmed.1000133

**Published:** 2009-09-29

**Authors:** Louisa Degenhardt, Wayne D. Hall, Michael Lynskey, John McGrath, Jennifer McLaren, Bianca Calabria, Harvey Whiteford, Theo Vos

**Affiliations:** 1 National Drug and Alcohol Research Centre, University of New South Wales, Sydney, Australia; 2 School of Population Health, University of Queensland, Herston, Australia; 3 Department of Psychiatry, Washington University School of Medicine, St. Louis, Missouri, United States; 4 Queensland Brain Institute, University of Queensland, St. Lucia, Australia; 5 Queensland Centre for Mental Health Research, University of Queensland, St. Lucia, Australia

## Abstract

Louise Degenhardt and colleagues discuss the evidence and the debate about whether Global Burden of Disease estimates should include cannabis use as a risk factor for psychosis.

**Summary**

- Comparative risk assessments estimate the proportion of a disease that can be attributed to a particular risk exposure and are important guides for health planning.
- In observational studies, there has been consistent evidence that cannabis use is associated with an increased risk of schizophrenia and more generally, psychosis.
- There is debate about whether such observational evidence is sufficient to infer that cannabis use is a contributory cause of psychosis.
- Given the controversy, should the comparative risk assessment in the current revision of the Global Burden of Disease (GBD) include an attribution of psychosis to cannabis use?
- We argue that the risk assessment should be included because the evidence is as good as that for many other risk factors included in the GBD, psychotic disorders are associated with substantial unavertable disability, and cannabis use is a potentially preventable exposure.

## Introduction

Evidence has accumulated suggesting that regular cannabis use is associated with psychotic symptoms and disorders in the general population [1],[2] and elevated among incident cases of psychosis [3],[4]. In this paper, we present the arguments for, and implications of, considering cannabis use as a risk factor for psychosis in the 2005 Global Burden of Disease (GBD) project.

## Examining Risk Factors for Disease Burden

Governments, policymakers, and funders need information on the comparative population health impact of different diseases and risk factors when making decisions about where to focus policy, services, and research. This field was revolutionised when the World Bank provided estimates using the disability-adjusted life year (DALY) [5]. This measure combined measures of premature mortality (years of life lost [YLL]) and morbidity (years lived with disability [YLD]) in order to estimate GBD. Estimates of burden attributable to various risk factors—“comparative risk assessment” (CRA) exercises [6]—are particularly important because they quantify and allow comparison of the extent to which reduction or removal of exposure to risk factors would reduce disease burden by using a measure of estimated Population Attributable Risks (PAR). The GBD uses fairly standard criteria to evaluate “risk factors”, in line with Bradford Hill's [7] oft-quoted criteria (Box 1).

**Box 1. Risk Factor Definitions in the 2005 GBD Project [66]**

**The GBD defines risks according to the following considerations:**

- Risk factors should be potentially modifiable;
- Risks should be assessed irrespective of place in a causal chain or scientific discipline that has traditionally analysed the risk factor, as long as evidence of causal effect can be established;
- Risks are defined to be not too broad (e.g., diet or environment as a whole) or too narrow (e.g., every single fruit and vegetable or every toxicant in tobacco smoke) with a relatively specific definition of risk factor exposure;
- Protective as well as hazardous factors are considered. However, the absence of a specific intervention should not be assessed as a risk factor, but rather in measurement of intervention coverage and effectiveness; and
- There exist sufficient data on risk factor exposure and risk-factor disease relationships.

## Evidence on the Association between Cannabis Use and Psychosis

In the previous global CRA, cannabis use was not included as a risk factor for any disease because of concerns about the quality of the evidence [8]. In the intervening years there has been a steady increase in the number and quality of research studies that have been conducted exploring the links between cannabis use and psychosis. Overall, these studies indicate that chance is an unlikely explanation of their association [9]–[11]. Recent reviews of prospective general population studies of associations between cannabis use and later psychosis (Table 1) [10],[11] concluded that although control for confounding reduced the size of the association, there was an increased risk of psychotic outcomes in individuals who used cannabis, with the greatest risk among those who used cannabis most frequently.

**Table 1:** Summary of two systematic reviews investigating cannabis use as a risk factor for psychosis.

Study	Details	Adjusted Pooled Estimate (95% CI)
Moore et al. [11]	Searched Medline, Embase, CINAHL, PsycINFO, ISI Web of Knowledge, ISI Proceedings, ZETOC, BIOSIS, LILACS, and MEDCARIB from their inception to September, 2006; searched reference lists of studies selected for inclusion; contacted experts. Studies were included if longitudinal and population based. Seven studies were included (some multiple papers). Data extraction and quality assessment were done independently and in duplicate.	Ever use: 1.41 (1.20–1.65)
		“Heavy” use: 2.09 (1.54–2.84)
Arsenault et al. [12]	The research strategies used were: computerized Medline and PsycLIT searches; cross-referencing of original studies; contact with other researchers in the field. Studies that included a well-defined sample drawn from population-based registers or cohorts and used prospective measures of cannabis use and adult psychosis.	2.34 (1.69–2.95)

It is useful to distinguish two primary ways in which cannabis use could be a “cause” of psychosis [12]. The strongest form of causal link is that heavy cannabis use causes a psychosis that would not otherwise have occurred. A second hypothesis is that cannabis use is a contributory cause: it might precipitate psychosis in vulnerable individuals—that it is one factor among many (including genetic predisposition and other unknown causes) that act together to cause psychotic disorders.

The evidence suggests that it is more likely that cannabis use precipitates psychosis in vulnerable persons, which is consistent with other lines of evidence suggesting that there is a complex constellation of factors leading to the development of psychosis (the stress-diathesis model of schizophrenia) and with studies suggesting that gene-environment interactions may provide some explanation of the association [13]. It is also consistent with conflicting evidence to date on whether changes in cannabis use have been associated with changes in the incidence of psychotic disorders in the general population [14]–[16].

There is also some evidence that cannabis use is associated with increased likelihood of relapse to psychosis among those who have developed a psychotic disorder [17], although the quality of control for confounding in these studies is poor [17]. In some studies cannabis use has also been associated with a younger age of onset of psychosis [18], although control for confounding variables in these has also been poor.

## Is the Association Biologically Plausible?

The principal psychoactive ingredient of cannabis is delta-9-tetrahydrocannabinol (THC), which acts upon a specific cannabinoid receptor (CB_1_) in the brain [19]. Although historically the dopaminergic system has been considered to play an important role in psychotic disorders [20], there is increasing evidence that the cannabinoid system may also be involved [21]. Some studies have used animal models to explore the impact of THC and related compounds on brain function [21]–[24]. These results are also stimulating new preclinical research aimed at describing neurobiological mechanisms of action linking cannabis and outcomes of interest to schizophrenia [22]. Rodent models are being developed to examine the impact of THC exposure on pathways implicated in clinical schizophrenia [21].

## What Do We Mean by “Psychosis”?

### 

#### Transient cannabis-induced psychotic symptoms

It is possible that cannabis use might temporarily trigger some symptoms of psychosis among some users. Such symptoms are clinically (and significantly) distinct from a psychotic disorder such as schizophrenia.

Other drugs such as amphetamine have also been shown to have the potential to trigger psychotic symptoms among some users [25]. Double-blind provocation studies using intravenous THC and related compounds in healthy controls are providing insights into the neurobiological correlates of cannabis-related transient psychotic symptoms and neuro-cognitive impairments [26]–[29].

Several cross-sectional studies have examined the relationship between cannabis use and self-reported psychotic experiences or psychotic symptoms in the general population. All have found that cannabis use (or cannabis use disorders) were more common among people reporting such experiences; and these associations persisted after controlling for other variables [1],[2],[12],[30]. Although these findings provide important clues to the mechanisms of action linking cannabis use and persistent psychotic symptoms and/or clinical diagnoses, these outcomes are less of a concern for the research community.

It is not always clear whether the psychotic symptoms endorsed in studies assessing the relationship between cannabis use and “psychosis” occurred only in the context of cannabis intoxication, or whether the symptoms were a more distal outcome of previous cannabis use. For example, the Fergusson et al. study [31] assessed the relationship between psychotic symptoms in the past month with cannabis use in the past year. It remains possible that the psychotic symptoms endorsed may have been experienced only while intoxicated. The instruments used to measure psychotic outcomes in the Arsenault et al. [32], Henquet et al. [33], and van Os et al. [34] studies contain instructions not to include psychotic symptoms that only occur in the context of intoxication. Further, the authors of the Swedish conscript study [35] maintain that it is unlikely that substance-induced intoxication would have been misdiagnosed as schizophrenia. We turn now to the evidence relating to more persistent symptoms or disorders.

#### Schizophrenia and other psychotic disorders

In case-control studies [36],[37], patients with schizophrenia are more likely to use cannabis than other psychiatric patients or normal controls [38]. The prevalence of use in patients with schizophrenia has varied between studies but it is generally higher than rates in the general population [38],[39].

Cross-sectional community surveys of psychiatric disorders have also documented higher rates of substance use disorders among persons with schizophrenia [40]. Nearly half of the patients identified with schizophrenia in the US ECA study had a diagnosis of substance abuse or dependence (28% for an illicit drug disorder) [41],[42]. In an Australian population-based survey, 11.5% of those who reported that they had been diagnosed with schizophrenia met ICD-10 criteria for a cannabis use disorder in the past 12 mo, and 21.2% met criteria for an alcohol use disorder. After adjusting for confounding variables, those who met criteria for cannabis dependence were 2.9 times more likely to report that they had been diagnosed with schizophrenia than those who did not [1].

The first evidence that cannabis use may precipitate schizophrenia came from a 15-y prospective study of cannabis use and schizophrenia in 50,465 Swedish conscripts [43]. This study investigated the relationship between self-reported cannabis use at age 18 y and the risk of being diagnosed with schizophrenia in the Swedish psychiatric case register during the next 15 y. Those who had tried cannabis by age 18 y were 2.4 times more likely to receive a diagnosis of schizophrenia than those who had not. The risk of a diagnosis of schizophrenia was related to cannabis use in a dose-response way to the number of times cannabis had been used by age 18. Compared to those who had not used cannabis, the risk of developing schizophrenia was 1.3 times higher for those who had used cannabis one to ten times, three times higher for those who had used cannabis between one and 50 times, and six times higher for those who had used cannabis more than 50 times. These results remained after statistical adjustment for two variables that were related to the risk of developing schizophrenia (personal history of psychiatric disorder and parental divorce).

A number of longitudinal studies have since been reported that have all supported the findings of the Andreassen et al. study. Zammit et al. reported a follow up of the Swedish cohort study, reporting on risk over a 27-y follow up that covers most of the risk period for the onset of psychotic disorders in a cohort that was first studied when 18–20 y old [35]. This study improved on the earlier study in a number of ways. The psychiatric register provided more complete coverage of all cases diagnosed with schizophrenia; there was better statistical control of a larger number of potential confounding variables, including other drug use, IQ, known risk factors for schizophrenia, and social integration; the study distinguished between cases that occurred in the first 5 y of the study period and those that occurred more than 5 y afterwards in order to look at the possible role of a syndrome; and the study undertook separate analyses in those who only reported using cannabis at the initial assessment.

Zammit et al. [35] also found cannabis use at baseline predicted an increased risk of schizophrenia during the follow-up period. There was a dose-response relationship with frequency of use, which persisted after statistical control for confounders, including a history of psychiatric symptoms at baseline. The same relationships were observed in the subset of the sample who only reported cannabis use at baseline and among cases diagnosed in the first 5 y after assessment and for the subsequent 22 y.

Zammit et al.'s findings were consistent with those of a study conducted by Van Os and colleagues [34]. This was a 3-y longitudinal study of the relationship between self-reported cannabis use and psychosis in a community sample of 4,848 people in the Netherlands. Participants were assessed at baseline on cannabis and other drug use. Psychotic symptoms were assessed using a computerised diagnostic interview. A diagnosis of psychosis was validated in positive cases by a diagnostic telephone interview with a psychiatrist or psychologist. A consensus clinical judgement was made on the basis of the interview material as to whether individuals had a psychotic disorder for which they were in need of psychiatric care.

Van Os et al. replicated and extended the findings of the Swedish cohort in a number of important ways. First, cannabis use at baseline predicted an increased risk of psychotic symptoms during the follow-up period in individuals who had not reported psychiatric symptoms at baseline. Second, there was a dose-response relationship between frequency of cannabis use at baseline and risk of psychotic symptoms during the follow-up period. Third, the relationship between cannabis use and psychotic symptoms persisted when they statistically controlled for the effects of other drug use. Fourth, the relationship between cannabis use and psychotic symptoms was stronger for cases with more severe psychotic symptoms that were adjudged to need psychiatric care. Fifth, those who reported any psychotic symptoms at baseline were more likely to develop schizophrenia if they used cannabis than were individuals who were not so vulnerable.

A study by Henquet et al. [33] replicated the Swedish and Dutch studies in a 4-y follow up of a cohort of 2,437 adolescents and young adults between 1995 and 1999 in Munich. Their participants were assessed at baseline on cannabis use and psychotic symptoms using a questionnaire. Psychotic symptoms were assessed in early adulthood using the Composite International Diagnostic Interview. They found a dose-response relationship between self-reported cannabis use at baseline and the likelihood of reporting psychotic symptoms. As in the Dutch cohort, young people who reported psychotic symptoms at baseline were much more likely to experience psychotic symptoms at follow up if they used cannabis than were peers who did not have such a history.

Arseneault et al. reported a prospective study of the relationship between adolescent cannabis use and psychosis in young adults in a New Zealand birth cohort (*n* = 759). Participants were assessed intensively on risk factors for psychotic symptoms and disorders since birth [32], and psychotic disorders were conservatively assessed according to DSM-IV diagnostic criteria, with corroborative reports from family members or friends on social adjustment. They assessed psychotic symptoms at age 11 y before onset of cannabis use and distinguished between early and late onset of cannabis use. They also examined the specificity of the association between cannabis use and psychosis by conducting analyses of the effects of: (1) other drug use on psychotic symptoms and disorders; and (2) cannabis use on depressive disorders.

Arseneault et al. found a relationship between cannabis use by age 15 y and an increased risk of schizophreniform disorder by age 26 y. Controlling for other drug use did not affect the relationship. The relationship was no longer statistically significant after adjustment for reporting psychotic symptoms at age 11 y, which probably reflected the small number of psychotic disorders observed in the sample. The small number of cases also limited the ability of the study to examine predictors of psychotic disorders at age 26 y. The measurement of cannabis and other drug use was crude (viz, none, 1–2 times, and 3 or more times), although this was more likely to work against finding relationships.

There was also specificity in the effects of cannabis on schizophreniform disorder: there was no relationship between other drug use and psychotic disorders, and no relationship between cannabis use and depression. There was also an interaction between psychosis risk and age of onset of cannabis use, with earlier onset being more strongly related to psychosis. There was also the suggestion of an interaction between cannabis use and vulnerability, with a higher risk of psychosis among cannabis users who reported psychotic symptoms at age 11 y.

Caspi and colleagues subsequently used the cohort to examine an interaction between cannabis use and a functional polymorphism of the *COMT* gene that codes for dopamine in their effects on the risk of psychosis [44]. They found that the 25% of the cohort who were homozygous for the polymorphism and used cannabis were 10.9 times more likely to have developed a schizophreniform disorder than peers with the same polymorphism who did not use cannabis. In the absence of this polymorphism, young adults who used cannabis were not at any increased risk of psychosis.

Apart from clinical diagnoses, several longitudinal studies have also examined the relationship between cannabis use and subclinical (or isolated) psychotic symptoms. Fergusson, Horwood, and Swain-Campbell have reported a longitudinal study of the relationship between cannabis dependence at age 18 y and the number of psychotic symptoms reported at age 21 y in the Christchurch birth cohort in New Zealand [45]. They assessed cannabis dependence using DSM-IV criteria and psychotic symptoms were assessed by ten items from the SCL-90. Because this was a birth cohort that had been assessed throughout childhood and adolescence Fergusson et al. were able to adjust for a large number of potential confounding variables, including self-reported psychotic symptoms at the previous assessment, other drug use, and other psychiatric disorders. They found that cannabis dependence at age 18 y predicted an increased risk of psychotic symptoms at age 21 y (relative risk [RR] of 2.3). This association was smaller but still significant after adjustment for potential confounders (RR of 1.8). More recently, Fergusson and colleagues examined the association between cannabis and psychotic symptoms until age 25 y with the same cohort of young adults, using a more sophisticated structural equations modelling design that accounted for both observed and nonobserved confounding factors [31]. As with their earlier study, they concluded that the association between cannabis and psychosis did not appear to be explained by confounding factors, and that the direction of the association appeared to be from cannabis use to symptoms of psychosis rather than vice versa.

One study of high risk young people has failed to report an association between cannabis use and psychosis risk. This study identified 100 young people at “ultra high” risk for psychosis [46] because of family history or prodromal symptoms of psychosis (on the basis of one or more of the following: schizophrenia in a first degree relative; the presence of attenuated psychotic symptoms; or a brief limited psychosis) in whom 18% reported symptoms of cannabis dependence in the past year. They assessed whether cannabis users were more likely to develop psychosis in the following year, but did not find any association, regardless of the frequency of cannabis use.

Increasingly, researchers in the field of psychosis are examining the concept of psychotic spectrum features as risk factors for psychosis [47]. Recent work has found that these symptoms are common in the general population distribution and can persist over a 20-y period. Two major trajectories have been identified: persistent “schizophrenic nuclear symptoms” (which resemble psychosis) and persistent “schizotypal symptoms” (more closely resembling schizotypal personality disorder) [47]. Cannabis use during adolescence has been found to be associated with “high load” schizophrenia nuclear symptoms during adulthood—but not so for the schizotypal symptom cluster. More frequent cannabis use was more strongly associated with persistent high load symptoms for the entire follow-up period. These findings suggest that there may be different aetiological dimensions for these two symptom dimensions, with an interaction between biological vulnerability and unique psychosocial risk factors for each symptom cluster; limitations of the study included the small number of cases, the use of open ended interviews, and the use of multiple analyses.

## The Effects of Varying Outcome Measures

There are several major criticisms of the above evidence. The first concerns the varying outcome measures that different studies have used. These include “psychosis,” psychotic symptoms, and schizophreniform disorders diagnosed using psychiatric interviews and psychiatric case registers.

How should this affect confidence in the study findings? We suggest that they are less of an issue than they first appear. First, as noted above, there is a growing recognition that psychotic-like experiences can provide valuable clues with respect to underlying neurobiological mechanisms and shared risk factors for psychotic disorders. Categorical diagnostic criteria do not provide the final word on these disorders. The exploration of psychotic symptoms (or psychotic-like experiences) has become a very fertile area of research [48]. These studies have generally shown that persons with these symptoms have an elevated risk of being formally diagnosed with a psychosis later in life [49]. In the section below, we review recent data on this issue.

Second, most studies of the association (with sample sizes of 1,000–4,000) have very low statistical power for detecting any effect that cannabis use has on the risk of diagnosed psychotic disorders. More prevalent outcomes, such as subclinical psychotic symptoms, have provided greater power to examine associations.

Third, we would also argue that the persistence of a correlation between cannabis use and these variously measured outcomes is more suggestive of a robust relationship than the contrary. This is because the use of differing measures of varying predictive validity may be expected to attenuate rather than positively bias measures of association between cannabis use and psychosis. Finally, the Swedish and Dutch studies that have investigated diagnosed psychotic disorders have found the same associations as studies of psychotic symptoms.

## Temporal Relationship

Many prospective studies share the weakness that they cannot precisely specify the timing of first cannabis use and the onset of psychotic symptoms. Participants have usually been assessed once a year or less often and asked to retrospectively report their cannabis use during the past year(s). This assessment has often been in terms of the total number of times cannabis was used, or the number of times on average that cannabis was used each week or month. Nonetheless, there are multiple prospective studies of representative samples of the general population, all of which show that cannabis use at one point in time is associated with psychotic symptoms at a later one, even after using a range of controls for confounding and various statistical approaches to analysis.

Studies undertaking more temporally fine-grained measurements have provided results consistent with these cruder measurements. A French study using an experience sampling method [50] found a positive association between self-reported cannabis use and unusual perceptions, and a negative association with hostility, over periods of hours. In those with pre-existing psychotic symptoms, cannabis use was more strongly associated with strange impressions and unusual perceptions, and its use did not decrease feelings of hostility [50].

Another study, involving monthly assessment of psychotic symptoms and cannabis use over 10 mo among persons with psychotic disorders, similarly found that more frequent cannabis use in one month was related to increases in psychotic symptoms a month later [51]. These lines of evidence suggest that the temporal relationship criterion is satisfied.

## Has the Evidence Base Been Affected by Publication Bias?

Publication bias is a potentially more serious concern: If negative results have been withheld from publication then the consistent positive results would be far less impressive than they seem from the published systematic reviews [52]. This possibility was investigated by the authors of one systematic review who surveyed researchers in the area asking about any studies with negative results that had not been published [10]. They concluded that this was not a serious issue in this instance.

## Is There Residual Uncontrolled Confounding?

The most difficult task in drawing causal inferences from observational studies is excluding the possibility that the relationship between cannabis use and psychosis is due to other uncontrolled factors (e.g., other drug use, genetic predisposition to develop schizophrenia and use cannabis, or self-medication). This has led some to object to calculation of estimates of population attributable risk (PAR) because the adjusted estimates are modest (typically around 2–3), and so open to the alternative explanation of uncontrolled confounding. For example, some have suggested that the propensity to take risks and engage in socially disapproved behaviour may be a common cause of cannabis use and psychotic symptoms [53]. Fergusson et al. attempted to address these criticisms by using fixed effects regression models to adjust for all unmeasured confounders [31].

Some may argue that a causal inference demands evidence that the cessation of cannabis use reduces these risks, as the evidence of risk reversal on cessation in the British doctors' study strengthened the case for a causal relationship between cigarette smoking and lung cancer and other diseases [54]. However, even this evidence did not persuade some sceptics in the case of tobacco use, with some arguing (unconvincingly) that those at lowest risk of these adverse health outcomes found it easier to quit [55]. What was probably more important was the consilience of a complex array of different types of evidence that convinced most public health officials that cigarette smoking caused these diseases [56]. In recent years, the range of methodologies used to investigate the association between cannabis use and psychosis has increased and there appears to be a similar convergence of evidence that the association between the two is causal.

It is difficult to see more conclusive evidence being produced for cannabis as a contributory cause of psychosis, or for the results of such studies to be as convincing as the evidence from cigarette smoking. This is because: the relationship between cannabis and psychosis is not as strong as that between smoking and lung cancer; the prevalence of cannabis use is so much lower than that for smoking; and the outcomes of psychosis are not as easy to study as mortality was in the Doll and Hill follow up of the British doctors. How then can we resolve the uncertainty that remains?

Epidemiology is an imprecise science, and recent experience has taught us to be cautious in making causal inferences from observational studies [57],[58]. With respect to cannabis, the findings from prospective longitudinal studies may still be vulnerable to residual confounding. The best way to deal with both known and unknown confounding from interventions (e.g., HRT) is to conduct randomised controlled trials to explore the impact of an exposure on the health outcome of interest. Clearly, this strategy cannot be used to explore the association between cannabis and psychosis. The use of twin or sibling-pair analyses can reduce unmeasured residual confounding to a certain extent [59], but these studies will still be vulnerable to criticisms of incomplete control for confounding. We have no choice then but to make cautious inferences about the role of cannabis in psychosis on the basis of observational studies.

## The Importance of Population Attributable Risk

Calculation of a PAR is important to place the magnitude of the cannabis and psychosis association in a population health context. Arsenault et al. [11] concluded that elimination of all cannabis use would reduce the incidence of schizophrenia in the United Kingdom by approximately 8%, assuming that the relationship was “causal” in the sense that schizophrenia would not have occurred in the absence of cannabis use; Zammit et al. [35] similarly estimated that 13% of schizophrenia cases in Sweden were attributable to cannabis use.

Nonetheless, these PAR estimates must be heavily qualified. Risk models related to complex and heterogeneous syndromes like schizophrenia will never be fully specified. Further, standard PAR estimates cannot account for the possibility that cannabis has brought forward the age of onset in an individual who would have otherwise developed the illness at a later age without exposure to cannabis [60]. Notwithstanding these limitations, a PAR has utility from a public health perspective in that it combines information about exposure-risk effect size and the prevalence of the exposure and helps the research community to prioritise public health interventions, a central aim of the GBD exercise. Because it is unlikely that we will ever have fully specified models, we should use PARs cautiously and conduct sensitivity analyses to assess the effects of uncertainty in our estimates.

## Is It Premature to Suggest that Cannabis Is a Risk Factor for Psychosis?

Some commentators may well argue that it is premature to conclude that the relationships between cannabis use and psychosis are causal, which raises the question of what the standard of proof should be causal inference. Some may argue for “proof beyond reasonable doubt,” the standard implicitly used in the last iteration of the GBD [8]. It is rare, however, to meet this standard of proof for noncommunicable diseases other than smoking-related diseases. What has changed since the last iteration of the GBD? The broad approach to all risk factors has been to set the standard of proof at “more likely than not,” rather than “beyond reasonable doubt.” If the latter was the standard of proof, then no adverse health consequences of cannabis would be considered apart from dependence.

If we had treatments that resulted in complete, immediate, and sustained remission for all individuals who develop psychosis, then the role of cannabis as an aetiological agent may attract less attention. But schizophrenia remains a poorly understood group of disorders. Even our best treatments are suboptimal [61],[62]. In the absence of better treatments, the most effective way to reduce the disability associated with schizophrenia is to prevent its occurrence when we can [63]. Thus, when considering potential risk factors for schizophrenia, we argue that candidates that offer the opportunity for public health interventions should be accorded more attention (e.g., education about the potential risks of cannabis use). Even exposures that may account for a small attributable fraction of those with the disorder warrant scrutiny.

## A Way Forward

Making estimates of the proportion of psychoses attributable to cannabis will in effect provide worst case estimates of the burden of disease (BoD) attributable to cannabis if the critics are correct that uncontrolled confounding explains the relationships between cannabis use and psychosis. In Australia, for example, cannabis use was included as a risk factor in the Australian BoD study, assuming causal relationships for cannabis dependence, psychosis, suicide, and car crashes [64]. Even after assuming that these relationships were causal, cannabis was not a major contributor to disease burden in Australia, accounting for 0.2% of all disease burden, which amounted to 10% of the total burden attributable to all illicit drugs [65]. These estimates are important for public policy purposes, because failure to make them allows untested estimates to be offered in public policy debate.

In the GBD project, we are considering several possible ways in which cannabis and psychosis may be linked. A range of estimates will be made as follows: (1) a model that will assume greater disorder severity among those using cannabis regularly who have already developed the disorder; (2) a model that will assume the association reflects earlier onset of the disorder among those who would have developed it anyway; (3) a model that will assume reduced remission from schizophrenia once it has developed; and (4) a model that assumes increased incidence of schizophrenia.

It is important to consider the consequences of not estimating this risk. There will be a reduced public health, policy, or research imperative, since there will be no estimated burden. If we do attempt to estimate burden, future work will examine the accuracy of our estimates and refine them as evidence accumulates. Debates may emerge and (hopefully) improvements made as new evidence supports or challenges the assumptions made. Estimates made in GBD 2005 should be seen as a first step in a process that can and should be improved with new data and new insights, including work for future estimates of country and global disease burden.
